# Lipopolysaccharide-Induced Neutrophil Dysfunction Following Transjugular Intrahepatic Portosystemic Stent Shunt (TIPSS) Insertion is Associated with Organ Failure and Mortality

**DOI:** 10.1038/srep40157

**Published:** 2017-01-04

**Authors:** Jane Macnaughtan, Rajeshwar P. Mookerjee, Schalk van der Merwe, Rajiv Jalan

**Affiliations:** 1 Liver Failure Group, UCL Institute for Liver and Digestive Health, UCL Medical School, Royal Free Hospital, London, United Kingdom; 2 Laboratory of Hepatology, KU Leuven, Belgium and Department of Internal Medicine, Division of Liver and biliopancreatic disorders, KU Leuven, Leuven, Belgium

## Abstract

Systemic lipopolysaccharide (LPS) is implicated in increasing mortality in patients with alcoholic hepatitis but the underlying mechanisms are not well characterised. The objective of this study was to characterise neutrophil function, LPS and cytokine concentrations within the splanchnic circulation of alcoholic cirrhotic patients undergoing TIPSS insertion for variceal haemorrhage and correlate this with outcome. 26 patients with alcoholic cirrhosis and variceal haemorrhage were studied prior to and 1-hour after TIPSS insertion. Neutrophil function, LPS and cytokine concentrations were determined in arterial, hepatic venous (HV) and portal venous blood (PV). Significantly higher LPS concentrations and neutrophil reactive oxidant species (ROS) production were observed in PV vs HV blood. Cross-incubation of HV plasma with PV neutrophils resulted in reduced ROS production. Insertion of TIPSS was associated with a significant increase in arterial LPS concentrations and deterioration in neutrophil phagocytosis. Number of organ failures and arterial IL-6 concentrations at presentation were associated with increased mortality. The portal circulation has a distinct immunological milieu characterised by a pathological neutrophil phenotype and an anti-inflammatory cytokine profile associated with heightened LPS levels. TIPSS insertion renders this neutrophil functional defect systemic, associated with an increase in arterial LPS and a susceptibility to sepsis.

Variceal haemorrhage is a common and life-threatening complication of cirrhosis. A growing body of evidence suggests the presence of a dynamic, modifiable component of portal hypertension consequent upon LPS (LPS)-induced inflammation, typically observed in an acute context. In cirrhotic rats, administration of intra-peritoneal LPS (LPS) has been shown to increase portal pressure^1^ and toll-like receptor 4 deficient mice are protected from portal hypertension following bile duct ligation^2^. Selective intestinal decontamination is associated with an improvement in vascular resistance and reduction in portal pressure^3^.

The relevance of LPS-induced inflammation in the context of variceal bleeding is evidenced clinically by the observed reduction in mortality and re-bleeding rates following administration of prophylactic antibiotics^4^,^5^. The central role of inflammation-driven portal hypertension is further demonstrated by the observed decrease in hepatic venous pressure gradient (HVPG) in patients with severe alcoholic hepatitis treated with anti-TNF (Tumour Necrosis Factor) α therapy^6^. Furthermore, correlations between pro-inflammatory mediators C-reactive protein and IL-6 and portal pressure and mortality have been observed^7^,^8^,^9^. The influence of the resultant systemic inflammation on intra-hepatic resistance is most marked in patients with more advanced disease, in particular, acute on chronic liver failure (ACLF)^10^; yet little is understood regarding underlying mechanisms. More recently, LPS levels were shown to increase the mortality of patients with alcoholic hepatitis^11^ but the mechanisms of how LPS leads to increased mortality is unknown.

A previous study from this group^12^ described a significant deterioration in neutrophil ROS production following TIPSS insertion for refractory variceal haemorrhage suggestive of a differential portal and systemic immunological milieu. We have also shown that LPS may contribute to this neutrophil dysfunction supporting the hypothesis that the observed relationship between LPS levels and mortality may be due to its effect on neutrophil function. Insertion of TIPSS allows simultaneous access to the hepatic and portal veins and, the systemic circulation allowing sampling of these vascular beds. The primary objective of this study was to characterise the differential nature of the innate immune response and LPS concentrations within the splanchnic vascular beds to determine to what extent they are compartmentalised within the portal circulation and rendered systemic by TIPSS insertion. We studied alcoholic cirrhotic patients undergoing TIPSS insertion indicated for refractory variceal haemorrhage. Neutrophil function, LPS levels and cytokine profile were determined in the portal and hepatic veins pre-TIPSS insertion together with the effect of cross-incubation of hepatic or portal venous plasma on portal and hepatic venous neutrophil function. By sampling the arterial inflow, we were able to calculate fractional extraction of LPS by the liver and intestine to ascertain the contribution of these organ systems to LPS kinetics. A further objective of this study was to determine which of these laboratory or clinical variables have prognostic relevance in the context of refractory variceal haemorrhage. We therefore sought to determine the associations of pro-inflammatory indices and organ failure with 30, 60 and 90-day mortality in this patient cohort to ascertain the prognostic significance of these factors.

## Results

### Patient characteristics

Twenty-six patients with alcoholic cirrhosis underwent TIPSS insertion indicated for variceal haemorrhage refractory to standard endoscopic therapy were the subjects of this study. The patient characteristics at the time of TIPSS insertion are summarized in Table 1. The mean age of the patient cohort was 49.62 (±2.45) years with a male: female ratio of 16:10. The mean MELD score of the patients in this study was 21.46 (±1.40) with ascites present in 19 patients with a mean West Haven score of 1.62 (±0.18). The mean laboratory values were as follows: INR 1.89 (±0.08), bilirubin 111.60 (±15.39) μmol/L, creatinine 125.80 (±13.68) μmol/L and albumin 27.88 (±0.68) g/dL. Mean portal pressure gradients pre- and post-TIPSS insertion were 21.04 (±1.09) mm Hg and 9.58 (±0.34) mm Hg respectively. 13 out of 26 patients died during follow-up in the study. Cause of death was as follows: multiple organ failure (n = 5), sepsis (n = 4), HE (n = 3). No cause of death was documented in 2 patients. The mean time from TIPSS insertion to death was 88.38 (+/−13.89) days. The mean follow up was 248.9 (+/−23.36) days in the survivor cohort.

### LPS studies

**Figure 1:**
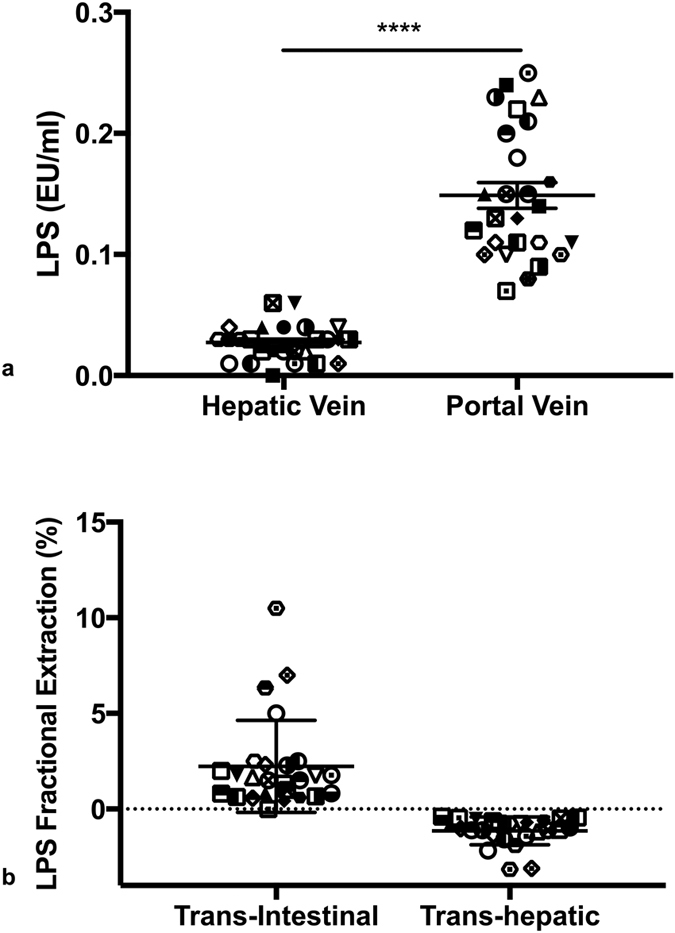
(**a**) LPS concentrations in the hepatic and portal venous plasma. Significantly higher endotoxaemia is observed in portal venous compared to hepatic venous plasma (****p < 0.0001) (**b**) Trans-Intestinal and trans-hepatic fractional extraction (FE) of LPS. Positive trans-intestinal LPS FE was observed consistent with gut-derived endotoxaemia. Negative trans-hepatic LPS FE was observed consistent with hepatic LPS clearance.

**Figure 2:**
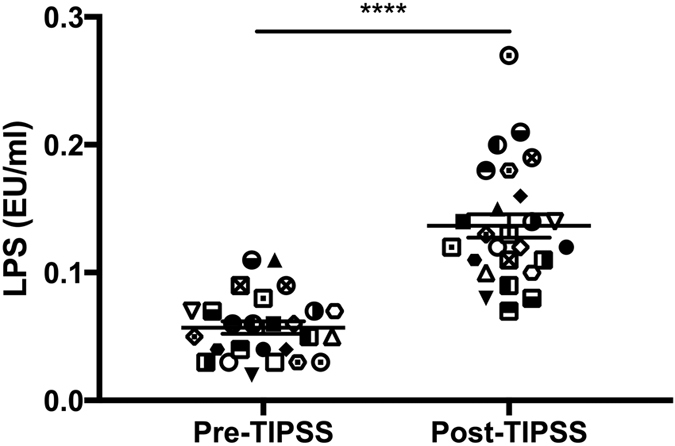
Arterial LPS concentrations pre- and 1 hour post-TIPSS insertion. Significant increases in arterial LPS concentrations are observed 1 hour post-TIPSS insertion (****p < 0.0001).

Mean LPS levels in the portal vein and hepatic vein pre-TIPSS insertion were 0.15 ± 0.01 and 0.03 ± 0.00 EU/ml respectively (p < 0.0001) (Fig. 1a). The pre-TIPSS trans-intestinal and trans-hepatic fractional extraction of LPS was observed to be 2.24 ± 0.47 and −1.14 ± 0.14 respectively (Fig. 1b). TIPSS insertion resulted in a significant increase in arterial LPS levels from 0.06 ± 0.00 to 0.14 ± 0.01 EU/ml (p < 0.0001) (Fig. 2).

### Neutrophil Function

**Figure 3:**
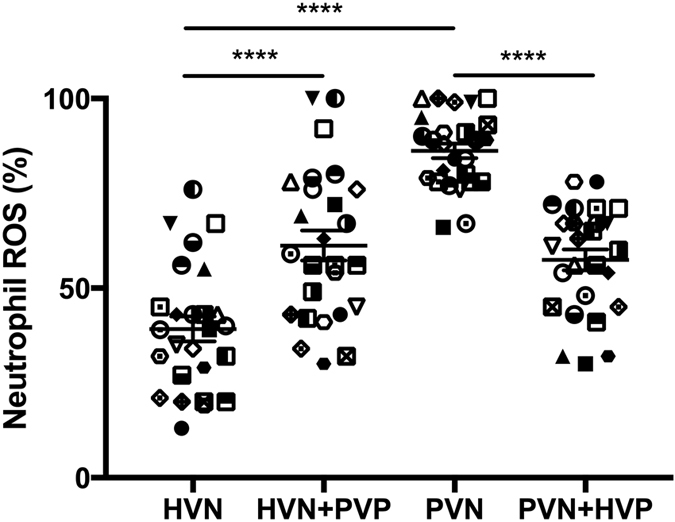
Reactive Oxidant Species (ROS) production in hepatic venous neutrophils (HVN) with and without co-incubation of portal venous plasma (PVP) and portal venous neutrophils (PVN) with and without co-incubation with hepatic venous plasma (HVP). Significantly higher ROS production was observed in PVN compared to HVN (****p < 0.0001). Co-incubation of HVN with PVP conferred a heightened ROS phenotype (****p < 0.0001). Conversely, co-incubation of PVN with HVP attenuated the heightened ROS phenotype (****p < 0.0001).

**Figure 4:**
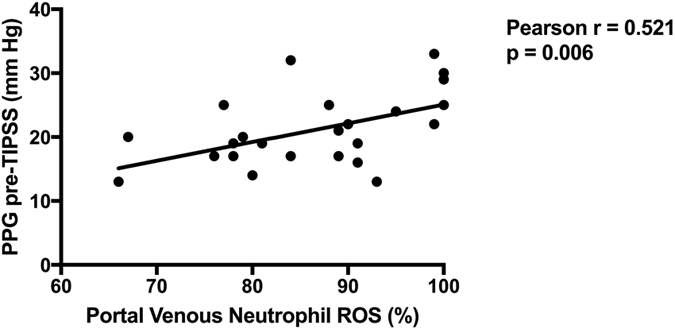
Correlation between Portal Pressure Gradient (PPG) pre-TIPSS and Portal venous neutrophil ROS production. A significant correlation between portal venous neutrophil ROS and portal pressure gradient prior to TIPSS insertion (Pearson r = 0.521, P = 0.006).

**Figure 5:**
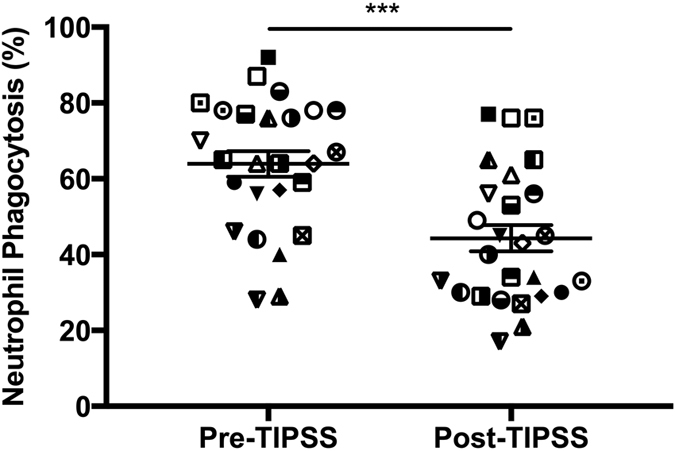
Arterial neutrophil phagocytosis pre- and 1 hour post-TIPSS insertion. Significant reductions in arterial neutrophil ROS production were observed 1 hour post-TIPSS insertion (***p < 0.001).

A significant difference in neutrophil resting burst was observed between hepatic and portal venous neutrophils (39.23 ± 3.28 vs 86.19 ± 1.92% respectively, p < 0.0001). Hepatic venous neutrophil resting burst was significantly increased from 39.23 ± 3.28 to 61.23 ± 3.95% after incubation with portal venous plasma (p < 0.0001) (Fig. 3). Conversely portal vein neutrophil resting burst was significantly reduced from 86.19 ± 1.92 to 57.46 ± 2.78% (p < 0.0001) after incubation with hepatic venous plasma. A significant correlation between portal venous neutrophil ROS and portal pressure gradient prior to TIPSS insertion was observed (Pearson r = 0.521, P = 0.006) (Fig. 4). Paralleling the observed increase in arterial LPS concentrations 1-hour post-TIPSS insertion, peripheral neutrophil function was significantly impaired following insertion of TIPSS. Neutrophil phagocytic function was significantly reduced from 63.92 ± 3.37% to 44.31 ± 3.47% (p = 0.0002) (Fig. 5).

### Cytokine studies

**Figure 6:**
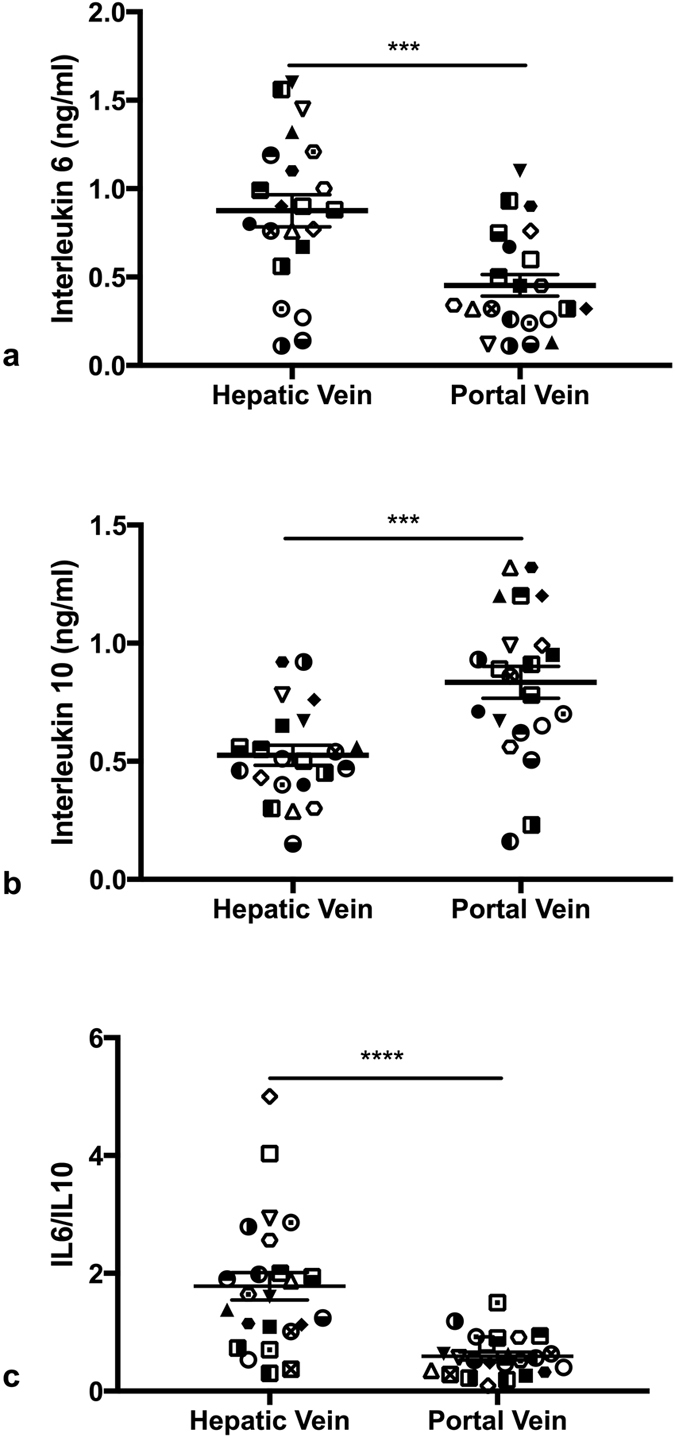
(**a**) Plasma interleukin 6 concentrations in the hepatic and portal veins (**b**) Plasma interleukin 10 concentrations in the hepatic and portal veins (**c**) Plasma interleukin 6/10.

Portal venous IL-6 levels were significantly lower than concentrations within the hepatic vein (0.45 ± 0.06 vs 0.88 ± 0.09 ng/ml) (p = 0.0004) (Fig. 6a). Conversely, IL-10 (Interleukin-10) levels were significantly higher in the portal vein compared to hepatic vein (0.83 ± 0.07 vs 0.53 ± 0.04 ng/ml) (p = 0.004) (Fig. 6b). IL-6/IL-10 ratios were significantly higher in the hepatic vein compared to portal vein (1.91 ± 0.26 vs 0.61 ± 0.08) (Fig. 6c). Arterial IL-6 concentrations were found to be predictive of mortality (supplemental data).

### Predictors of Survival

**Figure 7:**
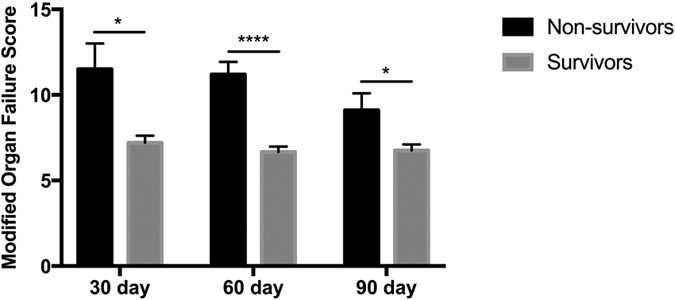
Mortality post-TIPSS insertion according to number of organ failures at the time of presentation.

Non-survivors were found to have significantly higher bilirubin and arterial IL6 compared to survivors. No significant difference in MELD score or portal pressure gradient were observed between the groups (Supplemental Figure). Increasing number of organ failures at presentation was associated with worsening 30, 60 and 90 day mortality (Fig. 7).

## Discussion

Variceal haemorrhage is a common complication of cirrhosis and associated with significant mortality. Clinically, the course may follow one of an acute uncomplicated decompensation event or a precipitant of ACLF. The pathogenic mechanisms underlying these clinical phenotypes remain poorly understood but an LPS-driven dysregulated inflammatory response is frequently observed and contributes to susceptibility to organ injury and immune paresis. Selective intestinal decontamination has been shown to confer survival benefit in this context implicating gut derived bacteria or bacterial products in pathogenesis^13^.

A significant LPS gradient was observed across the liver between portal and hepatic venous beds prior to TIPSS insertion associated with high positive trans-intestinal and negative trans-hepatic fractional extraction of LPS. This highlights that it is a defect at the gut-barrier interface, which predominantly contributes to systemic endotoxemia in refractory variceal haemorrhage and by implication constitutes the best target for therapy. Of note, the aetiology of cirrhosis in this cohort was exclusively alcoholic with no pre-determined period of abstinence. Patients with alcoholic cirrhosis have been shown to have significantly higher LPS concentrations than their non-alcoholic counterparts^14^. This is attributable to promotion of small intestinal bacterial overgrowth and direct toxic effects on the mucosa due to metabolites such as acetaldehyde^15^. Poor nutritional status may further compound this. Metabolic stressors relevant to refractory variceal haemorrhage such as oxidative injury and hypoxaemia may further contribute in the acute context.

In parallel to the finding of a trans-hepatic LPS gradient, we observed a ‘compartmentalization’ of neutrophil dysfunction, manifest by increased resting burst. Portal venous neutrophils were found to exhibit a pathologically heightened respiratory burst, which was significantly elevated compared to hepatic venous neutrophils, within a range known to be associated with excess mortality^16^. This neutrophil defect could be conferred or abrogated by co-incubation of portal venous or hepatic venous plasma respectively. Previous studies in patients with alcoholic cirrhosis demonstrated that a humoral factor, LPS, is responsible for mediating neutrophil dysfunction, a finding consistent with this data^16^. In these studies, neutrophil dysfunction, evidenced by both heightened ROS production and diminished phagocytosis, was normalised upon removal of LPS by either use of polymyxin B columns or anti-CD14 antibodies^16^. Further studies from our group have demonstrated an increased surface expression of TLR2 and 4 on the neutrophils of cirrhotic patients^17^ implicating LPS in pathogenesis. Of note, this study did not include a control population but is comparative to previous studies from our group by use of similar methodology.

Portal venous neutrophil ROS production was found to significantly correlate with portal pressure gradient. Portal hypertension is determined by both fixed and dynamic components of intrahepatic resistance, the latter of which is governed by vasomotor responsiveness and levels of vasoactive mediators. It is now understood that inflammation can modulate these variables. An increase in oxidative stress has been shown to result in reduced nitric oxide bioavailability^10^. It follows therefore that an increase in portal neutrophil-derived reactive oxidant species may result in lower portal nitric oxide levels with a resultant increase in portal pressure. Validation of these observations and mechanisms should be the focus of future studies.

TIPSS insertion was associated with a significant rise in arterial LPS concentrations and reduction in arterial neutrophil phagocytosis. Research from this group^12^ has previously identified TIPSS insertion to be associated with a significant increase in neutrophil ROS production associated with an increase in arterial LPS. This was correlated with an increase in whole body nitric oxide production and increased iNOS activity in HUVEC cell lines. Clinically, this was associated with a significant increase in cardiac output and cerebral blood flow.

IL-6/IL-10 ratios were calculated as a measure of the relative pro- or anti-inflammatory cytokine response. Hepatic venous and arterial ratios were found to be similar however ratios in portal venous blood are more skewed towards an anti-inflammatory response. Positive intestinal IL-10 fractional extraction was observed consistent with intestinal generation of IL-10. The most likely origin of portal-derived IL10 is from local innate immune populations and enterocytes. Indeed, increased interleukin-10 has been observed in the ileum of portal hypertensive rats^18^. Production of IL-10 is a key strategy lamina propria macrophages employ to induce an inhibitory, tolerising immunological phenotype in the gut in response to enterocyte stress. Metabolic stress is evident in intestinal mucosa even in the context of stable cirrhosis^19^. Portal hypertension with splanchnic venous stasis and associated hypoxia is a likely driver of this. Trafficking neutrophils may further contribute and indeed have been also shown to be significant IL-10 producers in the context of advanced cirrhosis^16^. Regulation of interleukin production within the mucosal-associated lymphoid tissue is complex and beyond the scope of this study.

In contrast to the intestine, the liver has a negligible role in IL-10 production but is a significant source of IL-6. Many different cell types within the liver including stimulated monocytes, fibroblasts and endothelial cells, generate IL-6. Hepatocytes may also be directly stimulated to produce IL-6 by factors such as bacterial LPS. Previous studies have demonstrated that IL-6 produced from Kupffer cells and hepatocytes following endotoxin challenge in alcoholic liver disease results in increased transcription of hepatoprotective pathways via STAT3, promoting hepatocellular proliferation in response to high portal venous endotoxin levels^20^.

This study highlights the prognostic relevance of organ failure and inflammatory indices in determining mortality following TIPSS insertion for refractory variceal bleeding. Conceptually, this reflects current understanding regarding the critical role of organ failure and a systemic inflammatory response syndrome in predicting outcome in advanced disease reiterating findings in large-scale studies^21^. Indeed, Ghaoui R. *et al*.^22^ identified that ACLF grade was predictive of mortality in a retrospective analysis of 239 patients with portal hypertension-related bleeding episodes.

In contrast, conventional measures of liver function such as MELD were not found to be predictive of survival in this context. This finding is in contrast with that of a recent study by Casadaban *et al*. of 101 patients undergoing TIPSS insertion for variceal haemorrhage^23^. This study identified MELD score to be predictive of 90 day mortality. Scores of >26, 19–25, 11–18 and < 10 were found to be associated with 90 day mortalities of 83%, 36%, 13% and 9% respectively. Potential factors which may account for the discordance in findings relate to the differences in patient population. Of note, the MELD scores in our patient population were high with a mean value of 21.46 ± 1.40 mm Hg. Lower MELD scores were not represented and the sample size is clearly much smaller than that of Casadaban *et al*. Conceptually the inability of MELD score to predict outcome may be due to other factors which govern survival not captured in the MELD score such as inflammatory indices, extra-hepatic/renal organ failure and regenerative capacity. Finally, our patient cohort were of exclusively alcoholic aetiology. Casadaban *et al*. described non-alcoholic aetiology as predictive of increased mortality on multivariate analysis^23^ which may be a function of diminished regenerative potential compared to patients with alcoholic cirrhosis.

Early TIPSS therapy for variceal haemorrhage in a carefully selected population, has been shown to be associated with a significant improvement in survival^24^. Other studies however have failed to demonstrate an improvement in survival despite an improvement in haemostasis rates^25^. Complications of TIPSS insertion in these populations included exacerbation of a hyper dynamic circulation, increased cerebral blood flow and the development of intracranial hypertension with increased encephalopathic grade^25^,^26^. Better patient selection and early prophylactic use of antibiotics may account for the discrepancies in findings between these studies. Our data implicates a gut-derived LPS-driven inflammatory response in pathogenesis and suggests a potential role for adjunctive LPS-reducing strategies to further diminish risk of organ failure and mortality induced by TIPSS.

## Conclusion

This study provides direct evidence for portal endotoxaemia in refractory variceal haemorrhage and describes the magnitude of bacterial translocation in this context. The data suggests that the liver is responsible for compartmentalising endotoxaemia and associated neutrophil dysfunction within the portal circulation but this aberrant neutrophil phenotype may be reversed by exposure to a different plasma milieu. TIPSS insertion disturbs this compartmentalisation and exposes the systemic circulation to portal endotoxaemia and a dysregulated inflammatory response syndrome, an adverse prognostic determinant in cirrhosis. This study also suggests an intestinal origin of IL-10 in cirrhosis, a possible adaptive response to enterocyte stress due to portal hypertension. The liver in contrast is a key generator of IL-6, a prognostic determinant in the context of variceal haemorrhage.

Number of organ failures at presentation was found to be associated with 30, 60 and 90 day mortality. LPS-driven SIRS, which drives multiple organ failure and sepsis is an important therapeutic target as evidenced by an improvement in mortality with prophylactic antibiotics. This study suggests that LPS-binding strategies, certainly in the initial period post-TIPSS insertion may improve neutrophil function and impact on organ failure and mortality. Early treatment of high-risk patients with adjunctive therapy to diminish portal endotoxaemia has the potential to diminish neutrophil dysfunction, risk of sepsis and survival in a patient group with an otherwise unacceptably high mortality.

## Methods

Studies were undertaken with the approval of Central London and Lothian Research Ethics Committees and in accordance with the Declaration of Helsinki (1951) of the World Medical Association.

### Patients

26 patients with alcoholic cirrhosis were studied prior to and 1-hour after TIPSS insertion. Data from 9 of these patients have been described in a previous study^12^. Patients were included into the study if they had clinical, biochemical or histological evidence of cirrhosis and were undergoing TIPSS placement indicated for active variceal haemorrhage refractory to endoscopic therapy and within 72 hours of admission. All patients were haemodynamically stable at the time of TIPSS insertion and informed consent (from the patient) or assent (from next of kin) was obtained. Other exclusion criteria included pregnancy, a diagnosis of diabetes, cardiovascular disease or malignancy.

### Management Protocol and Study Design

The patients were managed according to a standardized protocol, and in accordance with the UK guidelines for variceal hemorrhage. TIPSS insertion was indicated either because the patient had variceal bleeding refractory to endoscopic therapy or due to recurrent bleeding despite endoscopic therapy. At the time of TIPSS insertion, the patients were mechanically ventilated following sedation with propofol and paralysed with atracurium besylate (300–600 µg/kg/hr). Routine invasive and electrocardiographic monitoring was performed. Fluid resuscitation and red cell concentrate were administered in guidance with the central venous pressure, mean arterial pressure and the haematocrit. All the patients received broad spectrum prophylactic antibiotics as prophylaxis for variceal haemorrhage. No patients were on rifaximin therapy at the time of admission. Data regarding other antibiotic usage was not available. Blood glucose levels were maintained between 5–7 mmol/l.

### TIPSS procedure and catheter insertion

A single interventional radiologist on each site performed all the TIPSS procedures using standard techniques. Pre-TIPSS procedure, a femoral artery was cannulated using an 18 gauge needle (Vygon leader company, Ecoven, France). The right internal jugular vein was then punctured and a 10 F sheath (William-Cook, Bjaeverskov, Denmark) introduced. The right or middle hepatic vein was selected using a stiff hydrophilic guide wire (Terumo, Hatagaya, Tokyo, Japan) and a fine stylet was used to puncture a branch of the portal vein. Blood from the femoral artery (A), hepatic vein (HV) and portal vein (PV) sampled before insertion of the TIPSS. An angioplasty balloon catheter was then used to dilate the parenchymal tract prior to stent insertion (10 mm Wallstent, Schneider, Bulach, Switzerland). Portal venous and inferior vena caval pressures were recorded pre- and post-TIPSS insertion and the portal pressure gradient determined (portal venous pressure minus inferior vena caval pressure). One hour after TIPSS insertion blood was sampled from the following: femoral artery, hepatic vein (not involving TIPSS-shunt) and portal vein.

### Blood sampling and Measurements

#### Calculations

Intestinal and hepatic fractional extraction rates of LPS were calculated according to the following equation using pre-TIPSS plasma levels. The calculation of hepatic fractional extraction assumes a 70% contribution from the hepatic artery and 30% contribution from the portal vein in this patient cohort. Previous data supports this assumption.


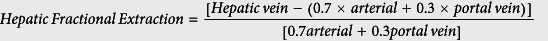



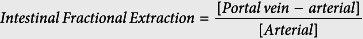


#### LPS

Heparinized whole blood was drawn with pyrogen-free needles into pyrogen-free tubes and the serum separated at 4◽C and stored at −80◽C in pyrogen-free polyethylene cryotubes (Nunc, Rochester, USA) on the day of collection. The chromogenic limulus amoebocyte lysate assay (Charles River Laboratories, France) was used for detection of LPS. For optimal test results, heparinised plasma with a low concentration of heparin (15IU/ml) was used, additionally plasma samples (100 μL) were diluted 1:10 with LPS-free water and heat treated for 30 minutes at 75 °C 100 μL of sample and 100 μL of LAL reagent were mixed in a 96-well plate and analysed at 405 nm with a spectrophotometer using the EndoscanV^®^ software.

### Neutrophil Studies

#### Neutrophil isolation

Whole blood was layered over 5 ml of Polymorphprep (Axis-Shield, Oslo, Norway) and spun for 30 minutes at 400 g at room temperature. Neutrophils were harvested from the second interface and washed with phosphate buffered saline (PBS, Sigma Aldrich, St. Louis, MO). Neutrophils were counted and re-suspended in PBS at a density of 5 × 10^5^ in 50 μL; 50 μL of cell suspension and 50 μL of plasma were used per assay. Viability was tested by Trypan blue exclusion and was over 98%.

#### Neutrophil function

The Phagoburst kit (Orpegen Pharma, Heidelberg, Germany) was used to determine the percentage of neutrophils that produce reactive oxidants as previously described (FACS Canto II, BD Bioscience) (Mookerjee, Stadlbauer *et al*.^16^). 100 μL of heparinized whole blood or blister neutrophil suspension was incubated for 20 minutes with either 20 μL of opsonized Escherichia coli (2 × 107 bacteria) or without stimulus at 37 °C. The formation of reactive oxidants was monitored by the oxidation of dihydrorhodamine 123 to rhodamine. Cells were stained with anti–CD16-PE antibody (Immunotools, Friesoythe, Germany) and analyzed by fluorescence-activated cell sorting. Neutrophils were gated using forward and side scatter and the percentage of CD16-positive cells producing reactive oxygen metabolites calculated.

The Phagotest (Orpegen Pharma) was used to measure phagocytosis by using FITC-labeled opsonized *E. coli* bacteria as described before (Mookerjee, Stadlbauer *et al*.^16^). 100 μL of either whole blood derived neutrophil suspension was mixed with 20 μL of FITC-labelled E. coli bacteria (2 × 10^7^) at 37 °C for 20 minutes. Fluorescence of bacteria at the cell surface was quenched using brilliant blue. To identify neutrophils, cells were stained with anti-CD16-PE immunoglobulin G (IOTest; Beckman Coulter) and analyzed by FACS analysis (FACS; Becton Dickinson FACS Canto II and FACS Diva 6.0 software, San Jose, CA). Phagocytosis is expressed as the geometric mean of fluorescence intensity (GMFI), corresponding to the number of labeled bacteria engulfed by a single cell.

##### Cytokine expression

Plasma levels of IL-10 and IL-6 were determined using commercially available ELISA assays in accordance with manufacturers instructions (R and D Biosystems).

##### Statistical Analysis

Statistical analysis was performed using Graph Prism Version 7.0. Parametric (paired t-test, Pearson) and non-parametric (Mann Whitney, Spearman) analyses were performed as appropriate. Significance was considered present at p < 0.05.

## Additional Information

**How to cite this article**: Macnaughtan, J. *et al*. Lipopolysaccharide-Induced Neutrophil Dysfunction Following Transjugular Intrahepatic Portosystemic Stent Shunt (TIPSS) Insertion is Associated with Organ Failure and Mortality. *Sci. Rep.* **7**, 40157; doi: 10.1038/srep40157 (2017).

**Publisher's note:** Springer Nature remains neutral with regard to jurisdictional claims in published maps and institutional affiliations.

## Supplementary Material

Supplementary Table 1

## Figures and Tables

**Table 1:** Patient characteristics at time of TIPSS insertion.

Patient Characteristics	Mean (SEM)
Age (yr)	49.62 (2.45)
Male:Female	16:10
MELD	21.46 (1.40)
Organ failure (N/Y)	
Liver	0/26
Kidney	14/12
Cerebral	0/26
Coagulation	0/26
Ascites	19
Laboratory data	
INR	1.89 (0.08)
Bilirubin (μmol/L)	111.60 (15.39)
Creatinine (μmol/L)	125.80 (13.68)
Albumin (g/dL)	27.88 (0.68)
Haemodynamic Data	
Pre-TIPSS PPG (mm Hg)	21.04 (1.09)
Post-TIPSS PPG (mm Hg)	9.58 (0.34)
