# Corrigendum: Lipopolysaccharide-Induced Neutrophil Dysfunction Following Transjugular Intrahepatic Portosystemic Stent Shunt (TIPSS) Insertion is Associated with Organ Failure and Mortality

**DOI:** 10.1038/srep45253

**Published:** 2017-04-28

**Authors:** Jane Macnaughtan, Rajeshwar P. Mookerjee, Schalk van der Merwe, Rajiv Jalan

Scientific Reports 7: Article number: 4015710.1038/srep40157; published online: 01 04 2017; updated: 04 28 2017

Schalk van der Merwe was omitted from the author list in the original version of this Article. This has been corrected in the PDF and HTML versions of the Article, as well as the Supplementary Information that now accompanies this Article.

The Author Contributions section now reads

“Rajiv Jalan and Schalk van der Merwe were responsible for project design. Rajiv Jalan was responsible for data collection. Data analysis, interpretation and writing of the article was performed by Jane Macnaughtan and Rajiv Jalan. Jane Macnaughtan, Raj Mookerjee, Schalk van der Merwe and Rajiv Jalan contributed to critical revision of the article. Jane Macnaughtan and Rajiv Jalan were responsible for producing the final version of the manuscript for publication”.

