# Erratum: Lipopolysaccharide-Induced Neutrophil Dysfunction Following Transjugular Intrahepatic Portosystemic Stent Shunt (TIPSS) Insertion is Associated with Organ Failure and Mortality

**DOI:** 10.1038/srep46990

**Published:** 2018-05-31

**Authors:** Jane Macnaughtan, Rajeshwar P. Mookerjee, Schalk van der Merwe, Rajiv Jalan

Scientific Reports 7: Article number: 4015710.1038/srep40157; published online: 01 14 2017; updated: 05 31 2018

In the Corrigendum published 28 April 2017, the author Schalk van der Merwe was incorrectly indexed. This error has now been corrected.

