# Supplementary material for: Lipopolysaccharide-Induced Neutrophil Dysfunction Following Transjugular Intrahepatic Portosystemic Stent Shunt (TIPSS) Insertion is Associated with Organ Failure and Mortality
Source: Sci Rep. 2017 Jan 4;7:40157. doi: 10.1038/srep40157 (PMC5209675; doi:10.1038/srep40157)
Supplement: Supplementary Table 1 [file srep40157-s1.doc]

**Lipopolysaccharide-Induced Neutrophil Dysfunction Following Transjugular Intrahepatic Portosystemic Stent Shunt (TIPSS) Insertion Is Associated With Organ Failure and Mortality**

**Authorship**

^1^Jane Macnaughtan, ^1^Rajeshwar P Mookerjee, ^1^Rajiv Jalan*

**Addresses**

^1^Liver Failure Group, UCL Institute for Liver and Digestive Health, UCL Medical School, Royal Free Hospital, London, United Kingdom

**Correspondence:**

Professor Rajiv Jalan. Liver Failure Group, UCL Institute for Liver and Digestive Health, UCL Medical School, Royal Free Hospital, London, United Kingdom

Phone: +442074332795; email: r.jalan@ucl.ac.uk

**Supplementary Table**

|  | **Survivor (Mean (SEM))** | **Non-Survivor (Mean (SEM))** | **p value** |
| --- | --- | --- | --- |
| Bilirubin (μmol/L) | 71 (8.693) | 152.2 (25.23) | **0.006** |
| INR | 1.78 (0.111) | 1.985 (0.124) | 0.236 |
| Albumin (g/dL) | 27.46 (1.035) | 28.31 (0.909) | 0.620 |
| Creatinine (μmol/L) | 99 (10.8) | 152.7 (23.31) | 0.062 |
| MELD | 19.46 (2.401) | 23.46 (1.304) | 0.156 |
|  |  |  |  |
| Portal Pressure Gradient Pre-TIPSS insertion (mm Hg) | 20.85(1.60) | 21.23 (1.53) | 0.969 |
| Portal Pressure Gradient Post-TIPSS insertion (mm Hg) | 9.308 (0.399) | 9.846 (0.553) | 0.437 |
|  |  |  |  |
| Arterial Neutrophil Phagocytosis (%) | 61.92 (4.747) | 65.92 (4.901) | 0.677 |
| Portal Venous Neutrophil ROS (%) | 87.77 (2.996) | 84.62 (2.545) | 0.292 |
| Hepatic Venous Neutrophil ROS (%) | 43 (4.633) | 35.46 (4.571) | 0.226 |
|  |  |  |  |
| Arterial Interleukin-6 (ng/ml) | 0.438 (0.082) | 0.782 (0.097) | **0.012** |
| Portal Venous Interleukin-6 (ng/ml) | 0.349 (0.076) | 0.578 (0.778) | 0.061 |
| Hepatic Venous Interleukin-6 (ng/ml) | 0.767 (0.135) | 0.984 (0.118) | 0.242 |
|  |  |  |  |
| Arterial Interleukin-10 (ng/ml) | 0.578 (0.103) | 0.654 (0.086) | 0.581 |
| Portal Venous Interleukin-10 (ng/ml) | 0.718 (0.119) | 0.891 (0.075) | 0.242 |
| Hepatic Venous Interleukin-10 (ng/ml) | 0.473(0.056) | 0.579 (0.717) | 0.216 |

**Table S1**

Clinical and immunological profiles of survivors and non-survivors
